# Regulation of Intrinsic and Bystander T Follicular Helper Cell Differentiation and Autoimmunity by Tsc1

**DOI:** 10.3389/fimmu.2021.620437

**Published:** 2021-04-14

**Authors:** Shimeng Zhang, Lei Li, Danli Xie, Srija Reddy, John W. Sleasman, Li Ma, Xiao-Ping Zhong

**Affiliations:** ^1^ Department of Pediatrics, Division of Allergy and Immunology, Duke University Medical Center, Durham, NC, United States; ^2^ Institute of Molecular Immunology, School of Laboratory Medicine and Biotechnology, Southern Medical University, Guangzhou, China; ^3^ Department of Breast and Thyroid Surgery, Union Hospital, Tongji Medical College, Huazhong University of Science and Technology, Wuhan, China; ^4^ Department of Microbiology and Immunology, School of Laboratory Medicine and Life Science, Wenzhou Medical University, Wenzhou, China; ^5^ Department of Immunology, Duke University Medical Center, Durham, NC, United States; ^6^ Hematologic Malignancies and Cellular Therapies Program, Duke Cancer Institute, Duke University Medical Center, Durham, NC, United States

**Keywords:** TSC1/2, mTOR, T follicular helper cells, germinal center B cells, autoimmunity, Regulatory T cells (T reg), T follicular regulatory (Tfr) cell

## Abstract

T Follicular helper (Tfh) cells promote germinal center (GC) B cell responses to develop effective humoral immunity against pathogens. However, dysregulated Tfh cells can also trigger autoantibody production and the development of autoimmune diseases. We report here that Tsc1, a regulator for mTOR signaling, plays differential roles in Tfh cell/GC B cell responses in the steady state and in immune responses to antigen immunization. In the steady state, Tsc1 in T cells intrinsically suppresses spontaneous GC-Tfh cell differentiation and subsequent GC-B cell formation and autoantibody production. In immune responses to antigen immunization, Tsc1 in T cells is required for efficient GC-Tfh cell expansion, GC-B cell induction, and antigen-specific antibody responses, at least in part *via* promoting GC-Tfh cell mitochondrial integrity and survival. Interestingly, in mixed bone marrow chimeric mice reconstituted with both wild-type and T cell-specific Tsc1-deficient bone marrow cells, Tsc1 deficiency leads to enhanced GC-Tfh cell differentiation of wild-type CD4 T cells and increased accumulation of wild-type T regulatory cells and T follicular regulatory cells. Such bystander GC-Tfh cell differentiation suggests a potential mechanism that could trigger self-reactive GC-Tfh cell/GC responses and autoimmunity *via* neighboring GC-Tfh cells.

## Introduction

T follicular helper (Tfh) cells are important players in both normal immune responses and autoimmune disease *via* contact-dependent and independent mechanisms to provide helping signals to B cells in the germinal centers (GCs). Tfh cells promote GC B cell proliferation and survival, Ig class switch and affinity maturation, and plasma cell and memory B cell formation (1, 2). However, deregulated Tfh cells can trigger abnormal GC B and memory B cell responses to produce autoantibodies and contribute to autoimmune diseases. Abnormal Tfh cells have been associated with or are the causal factors for autoimmune diseases in human patients and/or in autoimmune mouse models (3–9). Tfh cells express Bcl6, a transcription factor critical for their differentiation (10–13). Tfh cell differentiation is regulated *via* multiple mechanisms, including TCR signal strength and duration, costimulatory signaling such as CD28 and ICOS, and chemokine receptors such as CXCR5 (12, 14–22). Additionally, Tfh cells and GC B cells are suppressed by T follicular regulatory (Tfr) cells to prevent dysregulated antibody responses and autoimmunity (23–25). Recently, evidence has emerged that mTOR, which integrates TCR, costimulatory, cytokine, and metabolic signals (26–28), is crucial for Tfh cell differentiation, homeostasis, and function *via* signaling through both mTOR complexes 1 and 2 to regulate Bcl-6 expression and Tfh cell proliferation, survival, and metabolism (29–33), and it regulates Tfr cell differentiation and function (29, 30).

TSC1/2 are key regulators of mTOR signaling, inhibiting mTORC1 and, in certain instances, promoting mTORC2 activities. TSC1, TSC2, and TBC1D7 form the core of the TSC protein complex. TSC2 contains GAP activity for RheB to inhibit mTORC1 activation. TSC1 is crucial for TSC2 stability (34–36). Via tight control of mTOR, TSC1/2 regulate diverse processes such as cell metabolism, growth, proliferation, differentiation, quiescence, stemness, and autophagy and play important roles in many diseases (37, 38). Recent studies have revealed significant impacts of Tsc1 deficiency on immune cell development and function using mouse models with tissue-specific Tsc1 ablation. These studies have demonstrated that Tsc1 deficiency greatly affects hematopoietic stem cells, conventional T cells, regulatory T cells, iNKT cells, B cells, NK cells, macrophages (including M1/2 polarization), dendritic cells, and mast cells to influence both adaptive and innate immune responses, self-tolerance, and diseases (39–52). In T cells, Tsc1 has been found to be important for T cell homeostasis, quiescence, anergy, and effector and memory responses (39–45). However, the role Tsc1 plays in Tfh cells regarding controlling antibody responses has been unknown. In this report, we demonstrate that Tsc1 performs differential roles in Tfh cell differentiation in the steady state and during immune responses to immunization. In the steady state, Tsc1 inhibits Tfh cell differentiation, and T cell-specific Tsc1 deficiency causes spontaneous Tfh cell differentiation, leading to the accumulation of GC-B cells and the production of autoantibodies. In contrast, Tsc1 positively contributes to Tfh cell differentiation and antigen-specific antibody responses after immunization—at least in part by promoting Tfh cell survival *via* maintaining mitochondrial integrity and reducing reactive oxygen species. Additionally, Tsc1 deficiency not only intrinsically promotes Tfh cell differentiation but also extrinsically leads to bystander Tfh cell differentiation of WT T cells in the steady state. The discovery of bystander Tfh cell differentiation suggests potential mechanisms for the development of autoantibody and autoimmune diseases.

## Materials and Methods

### Mice

*Tsc1^f/f^* mice (53) and *Cd4-Cre* (54) mice were purchased from the Jackson Laboratory and Taconic Farms, respectively. *Tsc1^f/f^* mice were backcrossed to the C57BL/6 background for nine generations. All mice were generated and used according to protocols approved by the Duke University Institute Animal Care and Use Committee.

### Flow Cytometry and Antibodies

Single-cell suspensions from the spleen were stained for surface markers with appropriate fluorochrome-conjugated antibodies in PBS containing 2% FBS on ice for 30 min. Intracellular Bcl-6, cMaf, Foxp3, and Gata3 staining was conducted using the Invitrogen eBioscience Transcription Factor Buffer Set, and Ki67, iNOS, IgG1, and IgG2b staining was done using the BD Bioscience Cytofix/CytopermTM solution according to the manufacturer’s protocols. Cell death was determined by Live/Dead Fixable Violet Dead Cell Stain (Invitrogen, Carlsbad, CA) according to the manufacturer’s protocol. Fluorescence-conjugated anti-mouse CD4 (GK1.5), TCRβ (H57-597), CD8 (53-5.8), CD44 (IM7), CD62L (MEL-14), CD45.1 (A20), CD45.2 (104), B220 (RA3-6B2), CXCR5 (L138D7), PD-1 (RMP1-30), ICOS (C398.4A), GL7 (GL7), Fas (JO2), IgG1 (RMG1-1), IgG2b (RMG2b-1), CD93 (AA4.1), B220 (RA3-6B2), SLAM (A12 (7D4), ICAM1 (YN1/1.7.4), iNOS (W16030C), and PB-Annexin V were purchased from Biolegend (San Diego, CA). Anti-mouse Gata3 (L50-823), cMaf (T54-853), Streptavidin (BV711), and Ki67 were purchased from BD Biosciences. The anti-Bcl-6 (BCL-DWN) and anti-Foxp3 (FJK-16s) antibodies were purchased from eBioscience. Goat anti-mouse IgG (H+L) antibody (Alexa Fluor 568) for the detection of the anti-Ki67 antibody, anti-phospho-S6 S235/236 (cupl43k) antibody, and anti-phospho-Akt S474 (SDRNR) antibody were purchased from Thermo Fisher Scientific. Reactive oxygen species (ROS) were detected with 2’,7’-dichlorodihydrofluorescein diacetate (H2DCFDA, Thermo Fisher Scientific). Briefly, after cell surface staining with appropriate fluorochrome-conjugated antibodies, cells were resuspended in PBS and incubated with 1 μmol CM-H2DCFDA at 37°C for 30 min in the dark. After washing, the cells were gently resuspended in 10% FBS IMDM for flow cytometry. Mitochondrial membrane potential was detected with TMRM (Thermo Fisher Scientific). Cells were incubated in PBS containing 1 μmol TMRM at 37°C for 30 min followed by other cell surface markers staining at 4°C. Data were collected using a BD LSRFortessaTM cytometer (BD Biosciences) and analyzed using the FlowJo Version 9.9.6 software. **Supplementary Figure 1** illustrates the gating strategies to identify Tfh cells, Tregs, and non-Tfh cell conventional T cells.

### Glucose Uptake

One million splenocytes in PBS in a 96-well plate were incubated with or without 100 μM 2-Deoxy-2-[(7-nitro-2,1,3-benzoxadiazol-4-yl)amino]-D-glucose (2-NBDG, Thermo Fisher) at 37°C with 5%CO2 for 30 min. After being washed with pre-clod PBS, the cells were stained with proper antibodies and analyzed by flow cytometry.

### Western Blot Analysis

Western blot analysis was performed as previously described (26). Briefly, WT and Tsc1-T-KO CD4 T cells were sorted from splenocytes with a Moflo sorter (Beckman Coulter). Cell lysates of 1 x10^6^ cells in 1% Nonidet P‐40 Lysis solution (1% Nonidet‐40, 150 mM NaCl, and 50 mM Tris, pH 7.4) with freshly added protease and phosphatase inhibitors were subjected to immunoblotting analysis with indicated antibodies. Anti-Tsc1 (D43E2, #6935), anti-phospho-S6 S235/236 (2F9, #4856), anti-phospho-4E-BP1 T37/46 (236B4, # 2855), anti-4E-BP1 (53H11, #9644), and anti-Akt1 (C73H10, #2938) antibodies were purchased from Cell Signaling Technology.

### Immunization and Measurement of Antibody Responses

Mice were immunized with a single i.p. injection of 20 μg of 4-hydroxy-3-nitrophenylacetyl conjugated chicken gamma globulin (NP_17_-CGG, Biosearch Technologies) in alum, as previously described (55). Serum was collected pre-immunization and on day 7, 14, and 21 post-immunization. Appropriately diluted sera were added into 96-well plates precoated with 50 μl 2 μg/ml NIP_4_-BSA or NIP_26_-BSA in 0.1 M carbonate buffer (pH 9.0) at 4°C overnight. After incubation and multiple washes, HRP-conjugated goat anti-mouse IgM, IgG, IgG1, IgG2b, and IgG3 were used to detect NIP-specific IgM, IgG, and IgG subtypes.

### Serum Immunoglobulin Concentrations

One hundred μl of appropriately diluted sera from unimmunized mice was added into 96-well plates (Corning, New York, NY) precoated with anti-mouse Igκ and Igλ antibodies (2μg/ml; SouthernBiotech, Birmingham, AL) in 0.1 M carbonate buffer (pH 9.0) at 4°C overnight. IgM, IgG, IgG1, IgG2b, and IgG3 levels were detected with ELISA using HRP-conjugated goat anti-mouse total or Ig subtype antibodies. Relative levels of Ig were computed by OD450 values.

### Chimeric Mice

CD45.1^+^CD45.2^+^ WT mice in C57BL/6 background were irradiated with a single dose of (1,000 rad) and were intravenously injected with 1.0 x 10^7^ BM cells of a mixture of CD45.2^+^ *Tsc^1f/f-^* Cd4cre mice and CD45.1^+^ WT mice at 1:1 ratio 4 h after irradiation. Recipient mice were euthanized and analyzed 8 weeks later.

### Statistical Analysis

Data were presented as mean ± SEM and analyzed for statistical differences using the Prism 5/GraphPad software. Data with each experiment that contained a pair of test and control mice that were the same sex and age, were hosted in the same cage, and in most cases were also littermates were analyzed with a two-tailed pairwise Student *t*-test. A connection line in the scatterplots indicates a pair of test and control mice in each experiment. Data that did not fall into the aforementioned pairwise Student *t*-test criteria such as from experiments with two or more test or control mice in one experiment were analyzed by an unpaired Student *t*-test. P-values less than 0.05 were considered significant.

## Results

### Deficiency of Tsc1 in T Cells Caused Constitutive Tfh Cell Differentiation and GC B Cell Formation

**Figure 1:**
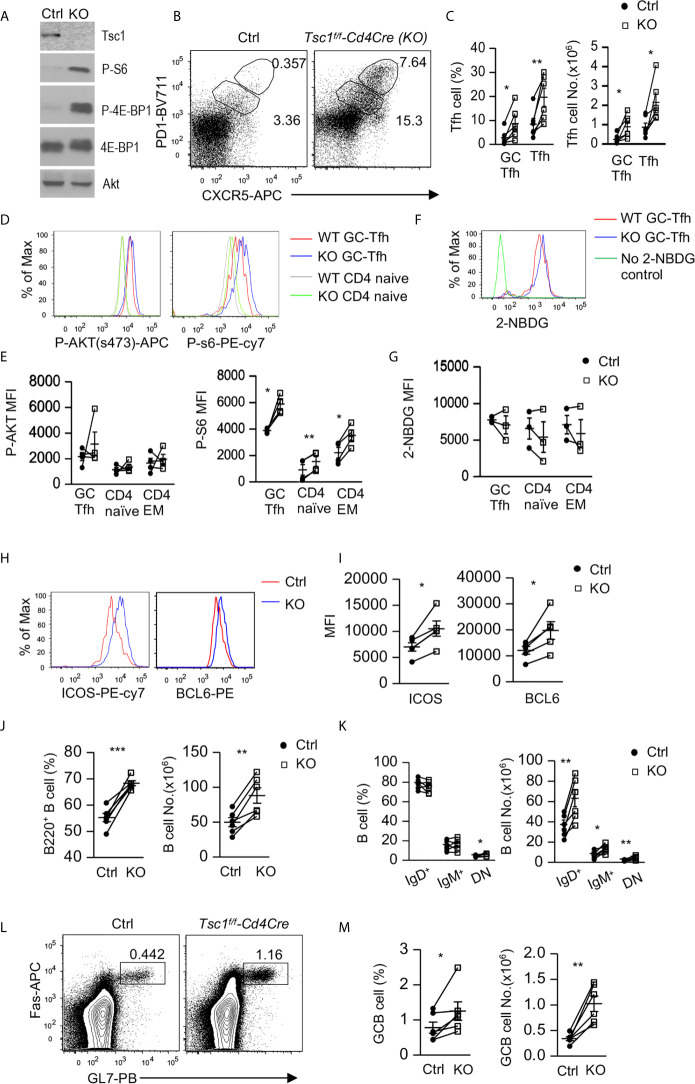
Inhibition of Tfh and GC responses by Tsc1. Splenocytes from 8–10 weeks old *Tsc1^f/f^Cd4Cre* and WT control mice were analyzed. **(A)** Immunoblotting of purified CD4 T cell lysates with the indicated antibodies. **(B)** Representative dot-plots of CXCR5 and PD1 staining in gated CD4^+^TCRβ^+^Foxp3^-^ T-cells. Gating strategies for GC-Tfh, Tfh, Treg, Tfr, naïve, CD44^+^CD62L^+^ central memory (CM), CD44^+^CD62L^-^ effector memory (EM) CD4 T cells are shown in **Supplementary Figure 1**. **(C)** Scatterplots represent mean ± SEM of GC-Tfh and Tfh cell percentages and numbers. **(D)** Overlaid histograms show Akt and S6 phosphorylation in CD4^+^TCRβ^+^Foxp3^-^ naïve and GC-Tfh cells. **(E)** Scatterplots represent mean ± SEM of mean fluorescence intensity (MFI) of phosphor-Akt and –S6 in CD4^+^TCRβ^+^Foxp3^-^ T cell populations. **(F)** Overlaid histograms show 2-NBDG uptake in GC-Tfh cells. **(G)** Scatterplots represent mean ± SEM of 2-NBDG MFI in CD4^+^TCRβ^+^Foxp3^-^ T cell populations. **(H)** Overlaid histograms show Bcl6 and ICOS levels in GC-Tfh cells. **(I)** Scatterplots represent mean ± SEM of Bcl6 and ICOS MFI in GC-Tfh cells. **(J)** Scatterplots show percentages and numbers of B220^+^ B cells. **(K)** Scatterplots show mean ± SEM of percentages and numbers of indicated B cell populations. **(L)** Representative dot-plots show GL7 and Fas staining in gated CD93^-^B220^+^ B cells. **(M)** Scatterplots represent mean ± SEM of GC-B cell percentages and numbers. Data represent or are pooled from 3–6 experiments. *p < 0.05; **p < 0.01, ***p < 0.001 determined by a pairwise two-tailed Student *t*-test.

To determine the role of Tsc1 in GC-Tfh cells, we analyzed *Tsc1^f/f^-Cd4Cre* (Tsc1-T-KO, KO) mice and *Tsc1^+/+^-Cd4Cre* or *Tsc1^f/f^* control (Ctrl) mice. In purified Tsc1-T-KO CD4 T cells, Tsc1 was virtually undetectable, but S6 and 4E-BP1 phosphorylation increased, indicating efficient ablation of Tsc1 and enhanced mTORC1 signaling in these cells (**Figure 1A**). In the spleen of unimmunized WT mice, few Foxp3^-^ CD4 T cells were CXCR5^+^PD-1^+^, markers for GC-Tfh cells, or CXCR5^int^PD-1^int^ (markers for Tfh cells). However, both Tfh cells and GC-Tfh cells were increased 5.8-fold and 2.7-fold in the percentages and 7.7-fold and 3.1-fold in the numbers, respectively, in Tsc1-T-KO mice (**Figures 1B, C**). Both mTORC1 and mTORC2 promote GC-Tfh cell differentiation in part by increasing glucose metabolism (26–28). In Tsc1-T-KO GC-Tfh cells and naïve and effector memory (EM) CD4 T cells, S6 phosphorylation was enhanced, suggesting enhanced mTORC1 activity; Akt phosphorylation at serine 374 was not obviously changed, suggesting normal mTORC2 activity (**Figures 1D, E**), and glucose uptake was not obviously changed (**Figures 1F, G**). The transcription factor Bcl6 and costimulatory molecule ICOS are critical for Tfh cell differentiation (10–13, 17, 19, 56, 57). In Tsc1-T-KO GC-Tfh cells, both Bcl-6 and ICOS levels were increased compared with WT GC-Tfh cells (**Figures 1H, I**). Together, these data suggest that TSC1 may negatively control mTORC1 and the expression of Bcl-6 and ICOS to prevent spontaneous GC-Tfh cell differentiation.

In Tsc1-T-KO mice, splenic B220^+^ B cell percentages and numbers increased (**Figure 1J**). Within B cells, IgM^+^IgD^-^ and IgM^-^IgD^+^ B cell percentages were not altered, but IgM^-^IgD^-^ (DN) B cell percentages increased about 30%. Due to increased total B cells, their numbers all increased (**Figure 1K**). Consistent with increased GC-Tfh and Tfh cells, Tsc1-T-KO mice also contained increased Fas^+^GL7^+^ GC B cells (**Figures 1L, M**). Thus, Tsc1 inhibited spontaneous Tfh differentiation and GC-B cell formation in the steady state.

### TSC1 Intrinsically Inhibited Tfh Cell Differentiation and Extrinsically Suppressed Bystander Tfh Cell Differentiation

**Figure 2:**
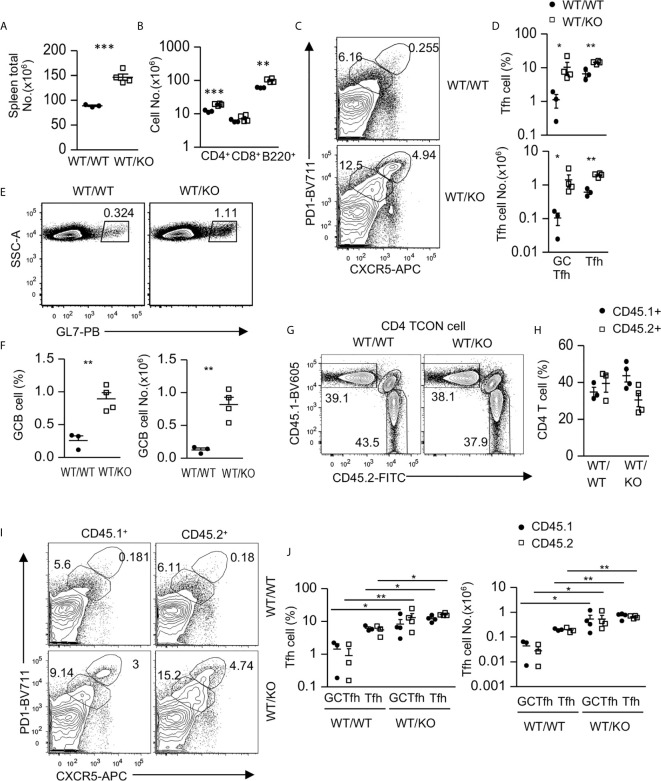
Intrinsic and extrinsic control of Tfh cell differentiation by Tsc1. CD45.1^+^CD45.2^+^ WT recipient mice were lethally irradiated (1,000 γ) and intravenously injected with a mixture of BM cells of either CD45.1^+^ WT and CD45.2^+^ WT (WT/WT) or CD45.1^+^ WT and CD45.2^+^ *Tsc1^f/f^-Cd4Cre* (WT/KO) BM cells at 1:1 ratio. Splenocytes in recipient mice were analyzed 6–8 weeks later. **(A)** Total spleen cell numbers. **(B)** Total CD4 and CD8 T cell and B cell numbers. **(C)** Representative contour-plots of CXCR5 and PD1 staining in gated CD4^+^TCRβ^+^Foxp3^-^T-cells. **(D)** Scatterplots represent mean ± SEM of GC-Tfh and Tfh cell percentages and numbers. **(E)** Representative dot-plots show GL7 staining in gated CD93^-^B220^+^ B cells. **(F)** Scatterplots represent mean ± SEM of GC-B cell percentages and numbers. **(G)** Representative contour-plots of CD45.1 and CD45.2 staining in gated CD4^+^TCRβ^+^Foxp3^-^ T (Tcon) cells. **(H)** Scatterplots show CD45.1^+^CD45.2^-^ and CD45.1^-^CD45.2^+^ CD4 T cell percentages. **(I)** Representative contour-plots of CXCR5 and PD1 staining in gated CD45.1^+^CD45.2^-^ and CD45.1^-^CD45.2^+^ CD4^+^TCRβ^+^Foxp3^-^ T-cells. **(J)** Scatterplots represent mean ± SEM of CD45.1^+^CD45.2^-^ and CD45.1^-^CD45.2^+^ GC-Tfh and Tfh cell percentages and numbers. Data represent or are pooled from three experiments. *p < 0.05; **p < 0.01, ***p < 0.001 determined by an unpaired two-tailed Student *t*-test.

Because Tsc1 was absent in both CD4 and CD8 T cells in *Tsc1^f/f^-Cd4Cre* mice, we generated mixed bone marrow (BM) chimeric mice to determine whether Tsc1 intrinsically controlled Tfh cell differentiation. We reconstituted lethally irradiated CD45.1^+^CD45.2^+^ recipient mice with a mixture of BM cells of either CD45.1^+^ WT and CD45.2^+^ WT (WT/WT) or CD45.1^+^ WT and CD45.2^+^ *Tsc1^f/f^-Cd4Cre* (WT/KO) BM cells at a 1:1 ratio. Six – eight weeks after reconstitution, WT/KO mice displayed enlarged spleens with increased total cell numbers compared with WT/WT mice (**Figure 2A**). Moreover, both CD4 T cell and B cell but not CD8 T cell numbers were increased in WT/KO mice (**Figure 2B**). Foxp3^-^ CD4 T cells in WT/KO mice had increased Tfh and GC-Tfh cell percentages and numbers (**Figures 2C, D**), accompanying increased GC B cells in both percentages and numbers (**Figures 2E, F**). Further analyses of CD45.1^+^CD45.2^-^ (CD45.1^+^) WT and CD45.1^-^CD45.2^+^ (CD45.2^+^, WT or Tsc1-T-KO) Foxp3^-^ CD4 T cells did not show significant differences between the ratios of CD45.1^+^ WT and CD45.2^+^ WT or Tsc1-T-KO Foxp3^-^ CD4 T cells in the chimeric mice (**Figures 2G, H**). Although CD45.2^+^ Tsc1-T-KO CD4 T cell percentages displayed a decreased trend compared with CD45.1^+^ WT CD4 T cells in the WT/KO mice, such differences were not statistically significant. Interestingly, both CD45.1^+^ WT and CD45.2^+^ Tsc1-T-KO Foxp3^-^ CD4 T cells had similarly increased Tfh and GC-Tfh cells in WT/KO mice compared with WT/WT mice (**Figures 2I, J**). The increases of CD45.1^+^CD45.2^-^ WT Tfh cells in WT/KO mixed chimeric mice were likely caused by positive feedback mechanisms from increased GC-B cells and/or by changes in the local environment caused by Tsc1-deficient CD4 T cells to favor Tfh differentiation. Together, these data suggest that Tsc1 exerted dual roles in inhibiting Tfh cell differentiation. On the one hand, it intrinsically inhibited Tfh cell differentiation. On the other hand, it extrinsically inhibited the bystander Tfh cell differentiation of neighboring CD4 T cells.

### Tsc1 Deficiency Resulted in Abnormal Tfh Cell Properties

**Figure 3:**
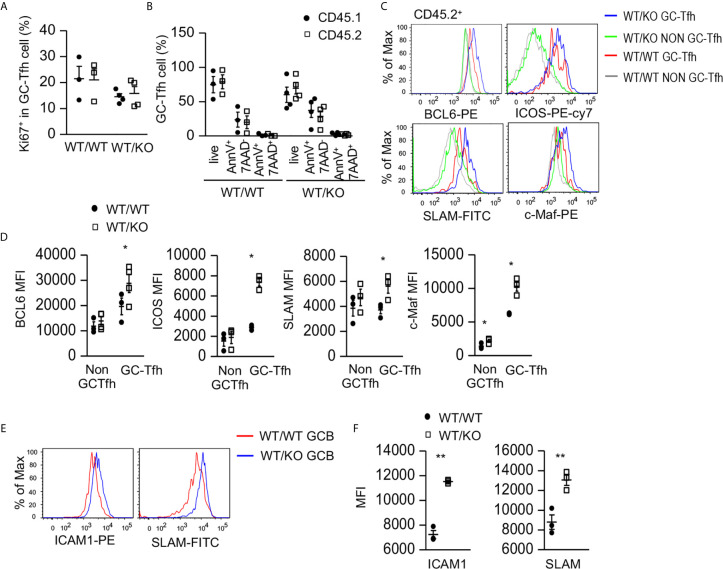
Increased expression of Tfh cell promoting molecules in Tsc1-deficient GC-Tfh cells. Splenocytes in the mixed BM chimeric mice shown in **Figure 2** were analyzed. **(A)** Scatterplots represent mean ± SEM of Ki67^+^ cells in CD45.1^+^CD45.2^-^ and CD45.1^-^CD45.2^+^ GC-Tfh cells. **(B)** Scatterplot represents mean ± SEM of survival and death rates of Tfh cells. **(C)** Overlaid histograms show Bcl6, cMaf, ICOS, and SLAM levels in GC-Tfh and non-GC-Tfh cells. **(D)** Scatterplots represent mean ± SEM of MFI of Bcl6, cMaf, ICOS, and SLAM in GC-Tfh and non-GC-Tfh cells. **(E)** Overlaid histograms show ICAM1 and SLAM levels in GC-B cells. **(F)** Scatterplots represent mean ± SEM of MFI of ICAM1 and SLAM levels in GC-B cells. Data represent or are pooled from three experiments. *p < 0.05; **p < 0.01 determined by an unpaired two-tailed Student *t*-test.

To examine how Tsc1 deficiency increased GC-Tfh cells, we examined GC-Tfh cell proliferation and survival in the mixed BM chimeric mice. Both CD45.1^+^ WT and CD45.2^+^ Tsc1-T-KO GC-Tfh cells in WT/KO mice and WT/WT mice expressed similar levels of Ki67, a marker of cell proliferation (**Figure 3A**) and showed a similar death rate (**Figure 3B**), suggesting that increased GC-Tfh cells in WT/KO mice was not due to increased proliferation or improved survival. Within CD45.2^+^ Tsc1-T-KO GC-Tfh cells, Bcl6, cMaf, ICOS, and SLAM levels increased (**Figures 3C, D**). Because these molecules promote Tfh cell differentiation and/or function (21, 58–60), Tsc1 may negatively control Tfh cell differentiation *via* the downregulating expression of these molecules. Interestingly, GC-B cells in WT/KO mice expressed higher levels of ICAM1 and SLAM than those in WT/WT mice (**Figures 3E, F**). Because ICAM1 interacts with LFA1 expressed on T cells to promote Tfh cell differentiation (61, 62) and SLAM strengthens GC B cell-Tfh cell interactions (63), GC-B cells with increased ICAM1 and SLAM in WT/KO mice might have enhanced capability to promote both Tsc1-T-KO Tfh cell and bystander WT Tfh cell differentiation.

### Elevated IgG1^+^ GC-B Cells and Serum IgG1 Autoantibodies in Tsc1-Deficient Mice

**Figure 4:**
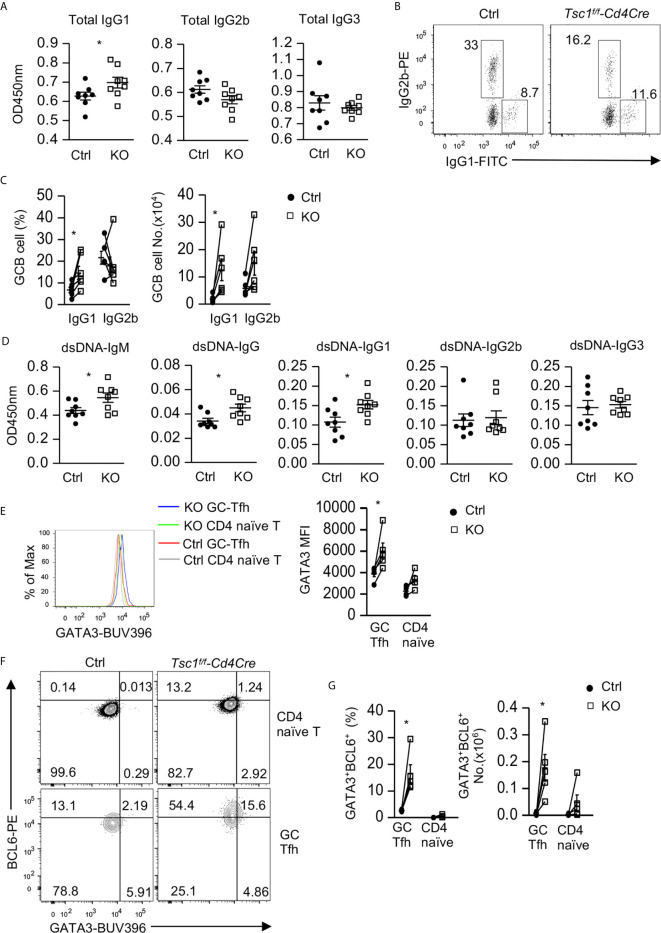
Elevated IgG1 humoral immunity and Tfh2 cells in Tsc1-deficient mice. **(A)** Serum concentrations of IgG subtypes. **(B)** IgG1 and IgG2b expression in GC-B cells from spleen. **(C)** Scatterplots show mean ± SEM of percentages and numbers of IgG1^+^ and IgG2b^+^ cells within GC B cells. **(D)** Serum autoantibodies levels detected by ELISA. **(E)** Overlaid histogram showed Gata3 levels in GC-Tfh and naïve CD4^+^TCRβ^+^Foxp3^-^ T cells. **(F)** Representative FACS plots show Gata3 and Bcl6 expression in GC-Tfh and naïve CD4^+^TCRβ^+^Foxp3^-^ T cells. **(G)** Scatterplots show percentages and numbers of Bcl6^hi^Gata3^+^ Tfh2 cells within GC-Tfh and naïve CD4^+^TCRβ^+^Foxp3^-^ T cells. Data shown are representative of or pooled from 2–5 experiments. *p < 0.05 determined by an unpaired **(A, D)** and pairwise **(C, E, G)** two-tailed Student *t*-test.

We next examined whether dysregulated Tfh/GC B cells in Tsc1-T-KO mice would lead to altered antibodies and enhanced autoimmunity. Tsc1-T-KO mice contained elevated serum IgG1 levels but normal serum IgG3 levels. Their IgG2b levels were slightly decreased, but such decreases were not statistically significant (*p* = 0.412, **Figure 4A**). Within splenic Tsc1-T-KO GC B cells, IgG1^+^ class-switched cells increased but IgG2 class-switched cells were not changed (**Figures 4B, C**), leading to increased numbers of IgG1^+^ cells but normal numbers of IgG2b^+^ cells (**Figure 4C**). Moreover, Tsc1-T-KO mice contained increased anti-double strand (ds) DNA IgM and IgG1but not IgG2b or IgG3 autoantibodies (**Figure 4D**). Interestingly, Tsc1-T-KO Tfh cells expressed elevated GATA3 levels (**Figure 4E**) and contained increased Bcl6^hi^GATA3^+^ Tfh2 cells (**Figures 4F, G**). Thus, TSC1 deficiency appeared to cause skewing of Tfh cells toward the Tfh2 cell sublineage, leading to elevated IgG1 class-switched GC B cells and serum IgG1 levels and the development of IgG1-dominant autoantibody responses.

### Intrinsic and Extrinsic Regulation of Treg and Tfr Cells by Tsc1

**Figure 5:**
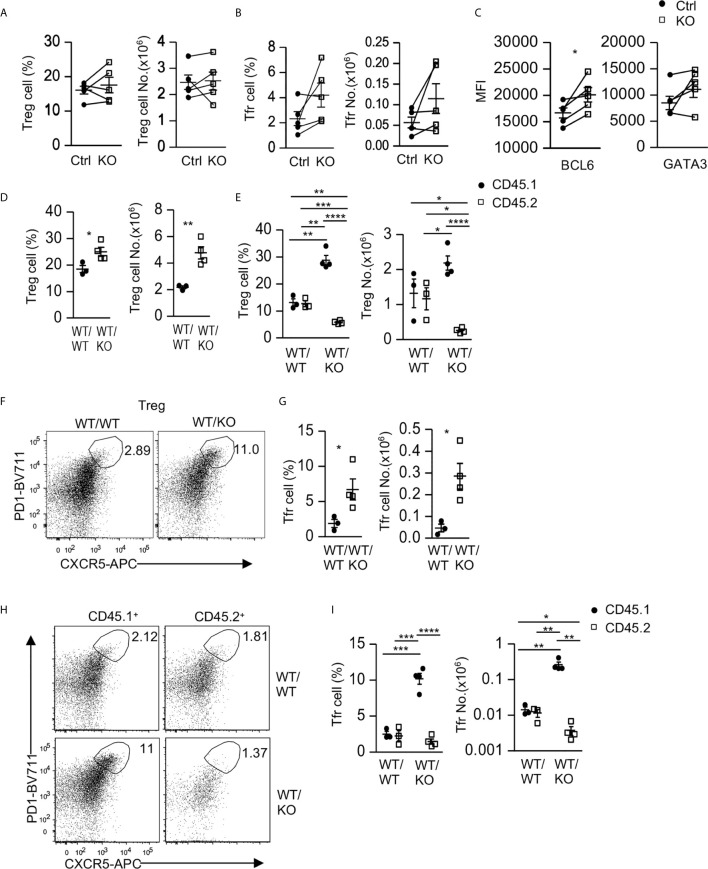
Intrinsic and extrinsic regulation of Tfr cells by Tsc1. **(A–C)** Splenocytes from 8–10 weeks old *Tsc1^f/f^Cd4Cre* and WT control mice were analyzed. **(A)** Scatterplots represent mean ± SEM of Treg percentages and numbers. **(B)** Scatterplots represent mean ± SEM of Tfr cell percentages and numbers. **(C)** Scatterplots represent mean ± SEM of Bcl6 and Gata3 MFI in Tfr cells. **(D–I)** Analysis of splenocytes in CD45.1^+^WT/CD45.2^+^WT (WT/WT) and CD45.1^+^WT/CD45.2^+^ Tsc1-T-KO (WT/KO) mixed BM mice described in **Figure 2**. **(D)** Scatterplots represent mean ± SEM of Treg percentages and numbers in WT/WT and WT/KO spleens. **(E)** Scatterplots represent mean ± SEM of CD45.1^+^ and CD45.2^+^ Treg percentages and numbers in WT/WT and WT/KO spleens. **(F)** Representative dot-plots of CXCR5 and PD1 staining in live gated splenic CD4^+^TCRβ^+^Foxp3^+^ Tregs in WT/WT and WT/KO mice. **(G)** Scatterplots represent mean ± SEM of Tfr cell percentages and numbers in WT/WT and WT/KO spleens. **(H)** Representative dot-plots of CXCR5 and PD1 staining in live gated CD45.1^+^ or CD45.2^+^ splenic Tregs in WT/WT and WT/KO mice. **(I)** Scatterplots represent mean ± SEM of CD45.1^+^ and CD45.2^+^ Tfr cell percentages and numbers in WT/WT and WT/KO spleens. *p < 0.05; **p < 0.01, ***p < 0.001, ****p < 0.0001 determined by a two-tailed pairwise **(A–C)** and unpaired **(D, E, G, I)** Student *t*-test.

Regulatory T cells (Tregs), especially Tfr cells, suppress Tfh/GC B cell differentiation and GC responses (23–25). mTOR regulates Treg and Tfr cell differentiation and function (29, 30, 64, 65). Although Tsc1 promotes Treg stability and function (51), its role in Tfr cells has been unknown. In *Tsc1^f/f^-Cd4Cre* mice, the percentages and numbers of Foxp3^+^ Tregs within CD4 T cells and CXCR5^+^PD1^+^ Tfr cells within Foxp3^+^ Tregs were not obviously different from WT mice (**Figures 5A, B**). However, Tsc1-T-KO Tfr cells expressed increased levels of Bcl6 but similar levels of Gata3 compared with WT Tfr cells (**Figure 5C**), suggesting that some properties of Tsc1-T-KO Tfr cells were altered. Given the dysregulated Tfh/GC B cell differentiation in Tsc1-T-KO mice, it is possible that Tsc1-deficient Tregs and Tfr cells might be functionally impaired to suppress GC-Tfh cell and GC B cell differentiation and/or that Tsc1-deficient GC-Tfh cells might be resistant to Treg/Tfr cell mediated suppression. Future studies should examine these possibilities.

Interestingly, in CD45.1^+^WT/CD45.2^+^WT (WT/WT) and CD45.1^+^WT/CD45.2^+^ Tsc1-T-KO (WT/KO) mixed BM mice, as described in **Figure 2**, Treg percentages and numbers in WT/KO mice increased compared with those in WT/WT mice (**Figure 5D**). Within WT/WT chimeric mice, Treg percentages and numbers in the CD45.2^+^ WT CD4 T cells and in the CD45.1^+^ WT CD4 T cells were similar (**Figure 5E**). In contrast, Treg percentages and numbers decreased within the CD45.2^+^ Tsc1-T-KO CD4 T cells but increased within the CD45.1^+^ WT CD4 T cells in WT/KO chimeric mice (**Figure 5E**). Such differences were also observed when they were compared with their counterparts in WT/WT chimeric mice. These data suggest that Tsc1 deficiency not only intrinsically limits Treg accumulation but also extrinsically promotes WT Treg accumulation in a competitive setting.

Within CD4^+^Foxp3^+^ Tregs, PD1^+^CXCR5^+^ Tfr cells were increased in WT/KO chimeric mice compared with WT/WT chimeric mice (**Figures 5F, G**). Moreover, the CD45.1^+^ WT Tfr cells increased, whereas the CD45.2^+^ Tsc1-T-KO Tfr cells decreased in both percentages and numbers within WT/KO chimeric mice. Such trends were also true when compared with their counterparts in WT/WT chimeric mice (**Figures 5H, I**). Thus, Tsc1 deficiency intrinsically inhibits Tfr cell differentiation/maintenance but appears to extrinsically promote bystander WT Tfr cell differentiation/maintenance.

### Impairment of Antibody Responses to Antigen Immunization in Tsc1-Deficient Mice

**Figure 6:**
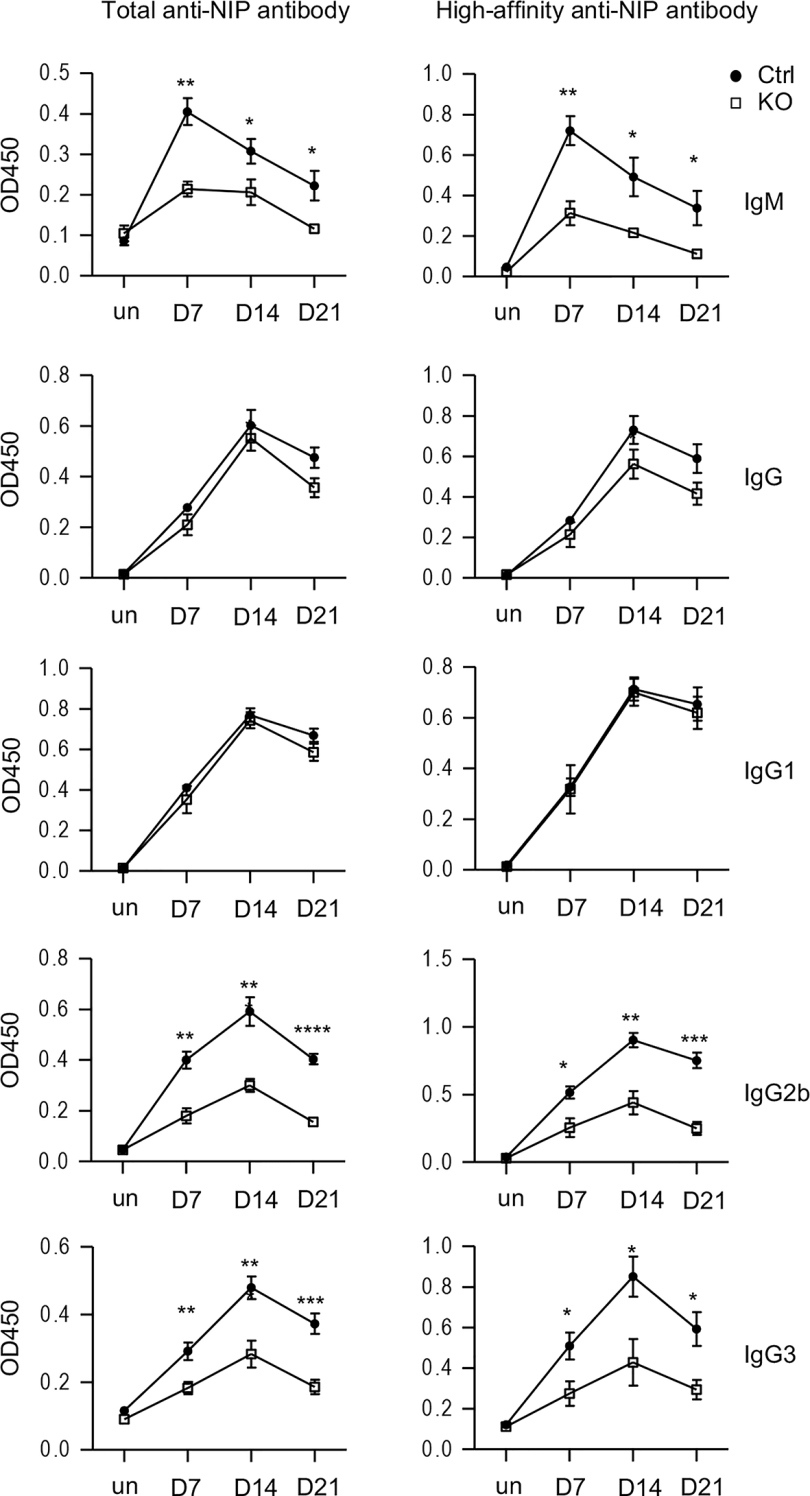
Impaired antigen-induced antibody responses in TSC1-deficient mice after immunization. *Tsc1^f/f^-Cd4Cre* and control mice were immunized with NP_17_-CGG in alum. Total and high affinity-anti-NIP antibodies before immunization and 7, 14, 21 days after immunization were measured *via* ELISA. Line plots show Mean ± SEM of serum concentrations of anti-NIP antibodies. Data shown are representative of two experiments. *p < 0.05; **p < 0.01; ***p < 0.001, ****p < 0.0001 determined by an unpaired two-tailed Student *t*-test.

We further asked whether Tsc1 deficiency in T cells would affect antigen-specific antibody responses after immunization. We immunized *Tsc1^f/f^-Cd4Cre* and *Tsc1^f/f^* or *Tsc1^+/+^-Cd4Cre* control mice with a T cell-dependent antigen NP-CGG (4-Hydroxy-3-nitrophenylacetyl hapten conjugated to chicken gamma globulin). In Tsc1-T-KO mice, both total and high affinity anti-NIP IgM titers decreased on days 7, 14, and 21 after immunization; although total and high affinity anti-NIP IgG titers displayed reduced trend, such decreases were not statistically significant (**Figure 6**). Further analyses of IgG subtypes revealed similar levels of IgG1 anti-NIP antibodies but obviously decreased IgG2b and IgG3 anti-NIP antibodies in Tsc1-T-KO mice compared with WT mice. Thus, TSC1 deficiency selectively impaired antigen-induced IgM, IgG2b, and IgG3 antibody responses with IgG1 responses spared.

### Impaired Tfh Cell Responses to Antigen Immunization in Tsc1-Deficient Mice

**Figure 7:**
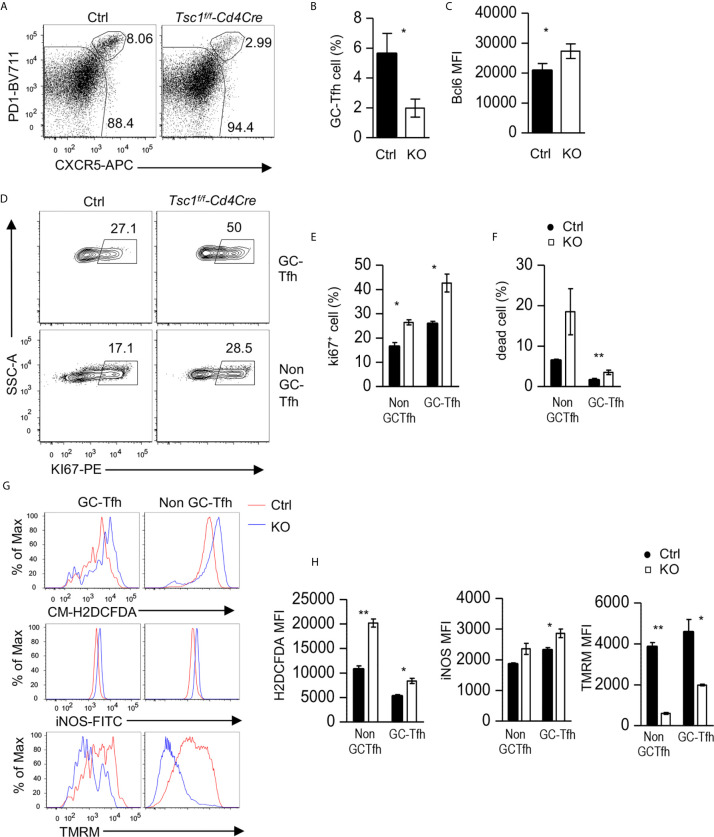
Impaired Tfh responses to antigen immunization in Tsc1-deficient mice. Splenocytes from *Tsc1^f/f^-Cd4Cre* and control mice 10 days after immunization with NP_17_-CGG in alum were analyzed. **(A)** FACS plots show CXCR5 and PD1 staining in live gated CD4^+^TCRβ^+^CD44^+^CD2L^-^ effective T cells. **(B)** Bar graphs represent mean ± SEM of GC-Tfh cell percentages. **(C)** Bar graphs represent mean ± SEM of MFI of Bcl6 in GC-Tfh cells. **(D)** Representative FACS plots show Ki67 staining in GC-Tfh and non-GC-Tfh cells. **(E)** Bar graphs represent the mean ± SEM of Ki67^+^ cells in GC-Tfh and non-GC-Tfh cells. **(F)** Bar graphs represent mean ± SEM of death rates of GC-Tfh and non-GC-Tfh cells. **(G, H)** Overlaid histograms show staining of CM-H2DCFDA (ROS), iNOS, and TMRM in GC-Tfh and non-GC-Tfh cells. **(H)** Bar graphs represent the mean ± SEM of MFI of H2DCFDA, iNOS, and TMRM in GC-Tfh and non-GC-Tfh cells. Data shown are representative of or pooled from three experiments. *p < 0.05; **p < 0.01, determined by an unpaired two-tailed Student t-test.

Given the observations of increased Tfh/GC B cells in *Tsc1^f/f^-Cd4Cre* mice in the steady state, it was surprising that antigen-specific antibody responses were impaired in these mice. We further examined GC-Tfh cells in these mice after NP-CGG/alum immunization. Splenic GC-Tfh cell percentages within CD44^+^ CD4 T cells decreased in Tsc1-T-KO mice compared with WT mice after immunization (**Figures 7A, B**). Tsc1-T-KO GC-Tfh cells expressed increased Bcl6 and Ki67 (**Figures 7C–E**), suggesting that their decreases were not caused by reduced Bcl6 or impaired proliferation. In contrast, the death rates of Tsc1-T-KO GC-Tfh cells and non-GC-Tfh CD4^+^ effector T cells were increased 2.9- and 2.4-fold, respectively, compared with WT controls (**Figure 7F**), suggesting that the increased death of Tsc1-deficient GC-Tfh cells might contribute to impaired GC-Tfh cells and antibody responses after immunization.

In both GC-Tfh and non-GC-Tfh cells from Tsc1-T-KO mice, reactive oxygen species (ROS) and iNOS levels increased, but mitochondrial potential, as reflected by TMRM (Tetramethylrhodamine methyl ester perchlorate) staining, decreased (**Figures 7G, H**). These data suggested that increased ROS, in the absence of Tsc1, might damage mitochondrial integrity and cause the death of GC-Tfh cells during immune responses to antigens.

## Discussion

Understanding the regulation of Tfh cell differentiation is important for developing new strategies to effectively elicit protective immunity and improve the treatment of autoimmune diseases. Previous reports have established the importance of Tsc1 for the tight control of mTOR in T cells to regulate multiple aspects of T cell functions. We demonstrate here that Tsc1 also plays differential roles in Tfh cell/GC-B cell differentiation in the steady state and in the response to immunization.

Our data indicate that Tsc1 intrinsically inhibits GC-Tfh cell and subsequent GC B cell differentiation and autoantibody production in the steady state. In Tsc1-deficient mice, GC-Tfh cells increased. Because Tsc1-deficient GC-Tfh cell proliferation and survival is similar to WT controls, Tsc1 deficiency is likely to augment CD4 T cell differentiation to GC-Tfh cells.

Because mTOR signaling is critical for GC-Tfh cell differentiation *via* multiple mechanisms such as differentiation, metabolism, survival, expansion, and migration (29–33) and is involved in T cell migration (66–68), and Tsc1 negatively controls mTORC1 signaling (30, 31, 33, 39–41, 69), Tsc1 may negatively control GC-Tfh cell differentiation *via* inhibiting mTOR signaling. Previous reports have found that PI3K/Akt signaling and its upregulation due to reduced or deficiency of PTEN promotes GC-Tfh cell differentiation (12, 17, 21, 70–72). Because the Tsc1/2 complex is inhibited by the PI3K/Akt pathway, our data are consistent with these observations and indicate that the PI3K/Akt/mTOR pathway needs to be tightly regulated at multiple steps for proper Tfh cell differentiation. In Tsc1-deficient GC-Tfh cells, Icos, SLAM, cMaf, and Bcl6 are upregulated, which may contribute to their enhanced differentiation. Engagement of ICOS with ICOSL expressed on B cells promotes GC-responses (73–75). Icos can signal through the PI3K-Akt-mTOR cascade to inactivate Foxo1 and promote Bcl6 and cMaf expression and GC-Tfh cell differentiation (12, 17, 18, 76, 77). cMaf intrinsically promotes Tfh cell differentiation and is important for promoting IL-21 expression to enhance GC-Tfh and GC B cell responses (12, 58, 59, 78). Although SLAM is not critical for GC-Tfh cell differentiation, it prolongs T cell–B cell contact for optimal Tfh cell function, especially expression of IL-4 and GC formation (60, 63, 79). In addition to SLAM, other SLAM family members such as CD84 and Ly108 regulate GC-Tfh cell differentiation and GC responses (79–81); it will be interesting to determine whether these molecules are affected by Tsc1 deficiency.

Our data also revealed that Tsc1 is required for optimal Tfh/GC B cell responses and antibody production to T-dependent antigens after immunization. The impaired antibody responses of Tsc1-T-KO mice to T-dependent antigens after immunization suggest that the proper control of Tsc1-regulated pathways is important for Tfh cell differentiation and function during antigen-induced responses. Tsc1-deficient Tfh cells contain increased ROS and manifested impaired mitochondrial integrity and increased cell death. It has been reported that CD44^+^ Tsc1-deficient CD4 T cells also contain high levels of ROS, and the treatment of these cells with a ROS scavenger improve their survival (39). It is plausible that Tsc1 prevents overproduction of ROS and subsequent mitochondrial damage to promote GC-Tfh cell survival during the immune responses to antigens. Additionally, Tsc1-deficient CD4 T cells are hyper-activated in the steady state (39–41), and excessive stimulation upon immunization could also contribute to impaired Tfh cell responses. Similar to GC-Tfh cells, Tsc1-deficient CD8 T cells are also defective in responses to microbial infection (43, 44). Of note, Tsc1 is deleted in naïve CD4 T cells in *Tsc1^f/f^-Cd4Cre* mice, and our data do not illustrate whether a Tsc1 deficiency affects multiple stages from T cell activation to GC-Tfh cell differentiation and homeostasis—or selectively affects GC-Tfh cells.

Our observations that Tsc1-deficient T cells enhance the bystander Tfh cell differentiation of WT CD4 T cells in mixed BM chimeric mice reconstituted with WT and *Tsc1^f/f^-Cd4Cre* BM cells are surprising and interesting because bystander Tfh cell differentiation has not been previously noted. Although the exact mechanisms that regulate bystander Tfh cell differentiation remain to be illustrated, there are multiple possibilities that Tsc1-deficient T cells, particularly Tfh cells, could cause bystander Tfh cell differentiation. Tsc1-deficient Tfh cells could lead to increased Tfh cell-promoting cytokines such as IL21 (82, 83) in the local environment to enhance bystander Tfh cell differentiation. They may also indirectly promote bystander Tfh cell differentiation *via* GC-B cells (84). Increased GC-B cell numbers and potentially altered properties associated with these B cells induced by Tsc1-deficient Tfh cells could promote WT T cell differentiation to Tfh cells and/or expansion of Tfh cells already generated in the steady state. GC-B cells in the WT/Tsc1-T-KO mixed BM chimeric mice expressed increased levels of ICAM1 and SLAM compared with those in WT/WT mice, which could not only positively provide feedback to Tsc1-deficient Tfh cells but also enhance WT Tfh cell differentiation/expansion as ICAM1 engages LFA1 on T cells and SLAM forms homodimer or heterodimer with SLAM on T cells to promote Tfh cell differentiation (61–63). Bystander Tfh cell differentiation could have important implications for autoimmune diseases. It is conceivable that bystander differentiation of self-reactive Tfh cells could occur during immune responses against pathogens or commensal bacteria, which could contribute to increased self-reactive Tfh cells and subsequent GC-B cells and subsequent autoantibody production. This hypothesis should be tested in the future.

In addition to Tfh cells, our data also indicate that Tsc1 is involved in the intrinsic and extrinsic regulation of Tfr cells. In *Tsc1^f/f^-Cd4Cre* mice, Tsc1 is not crucial for Tfr cell accumulation but negatively regulates Bcl6 expression. In mixed BM chimeric mice, Tsc1-deficient Tfr cells and Tregs are less competitive than their WT counterparts. The striking increases of WT Tregs, and especially Tfr cells in WT/KO mice, suggest that Tsc1 may extrinsically prevent bystander Treg and Tfr cell differentiation/accumulation. The increases of WT Treg and Tfr cells could be a compensatory response to the increased Tfh and GC B cells in these mice due to changes of the local environment that promote Treg and Tfr cell differentiation or both. Additional studies are needed to understand the mechanisms by which Tsc1 intrinsically and extrinsically regulates Tfr/Treg and Tfh/GC B cells.

## Data Availability Statement

The raw data supporting the conclusions of this article will be made available by the authors, without undue reservation.

## Ethics Statement

The animal study was reviewed and approved by Duke University Institute Animal Care and Use Committee.

## Author Contributions

SZ designed and performed experiments, analyzed data, and prepared the manuscript. LL participated in data analysis. DX and SR performed experiments and analyzed data. LM and JS participated in data interpretation and manuscript preparation. X-PZ conceived the project, designed experiments, and prepared the manuscript. All authors contributed to the article and approved the submitted version.

## Funding

Research in this manuscript was supported by the Institute of Allergy and Infectious Diseases of the NIH (R01AI079088) and a Translating Duke Health Pilot Project Grant in Immunology.

## Conflict of Interest

The authors declare that the research was conducted in the absence of any commercial or financial relationships that could be construed as a potential conflict of interest.
