# Supplementary figures and images for: Regulation of Intrinsic and Bystander T Follicular Helper Cell Differentiation and Autoimmunity by Tsc1
Source: Front Immunol. 2021 Apr 14;12:620437. doi: 10.3389/fimmu.2021.620437 (PMC8079652; doi:10.3389/fimmu.2021.620437)

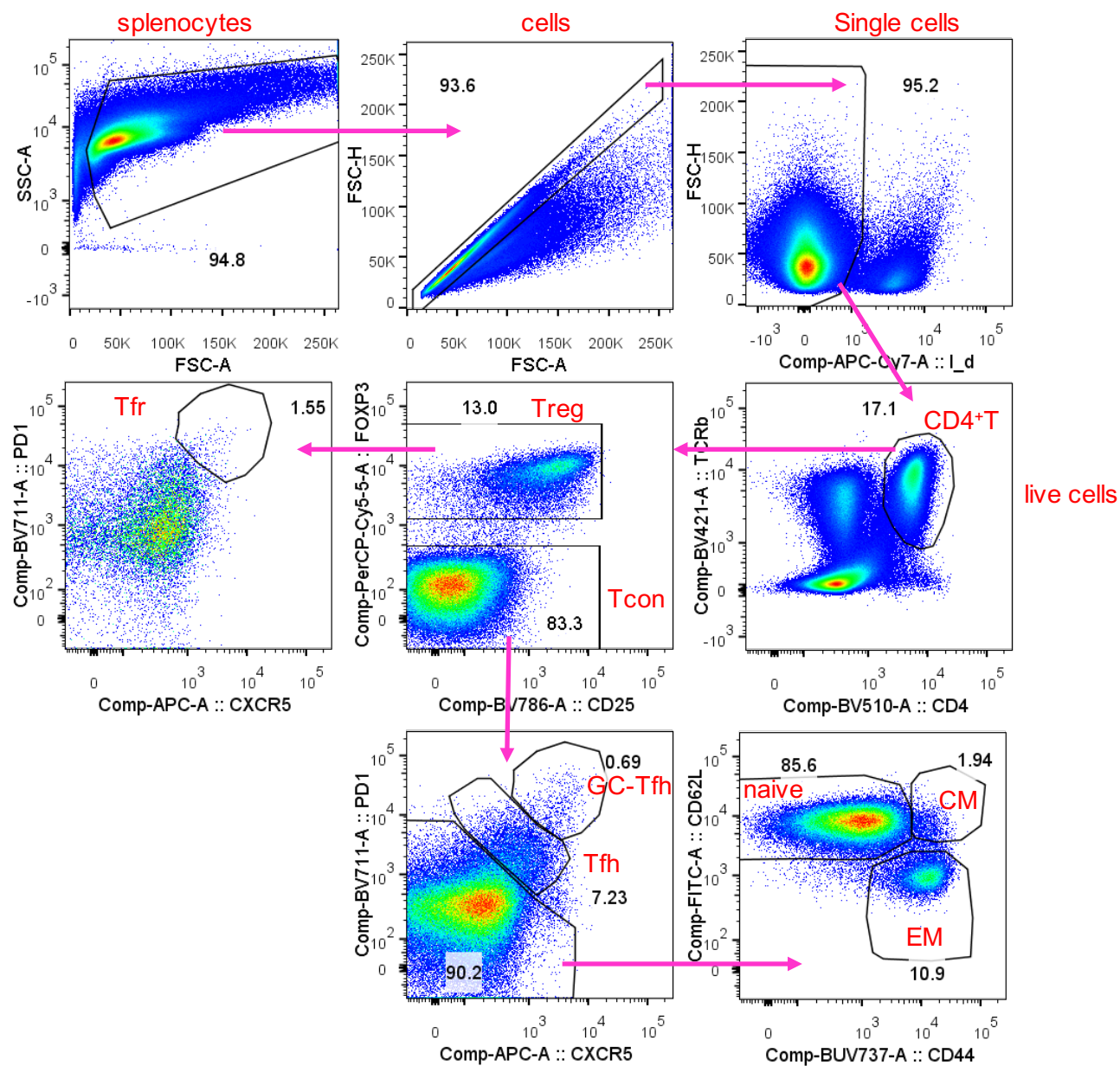

Supplementary Figure 1. Gating strategy of CD4 T cell populations in WT splenocytes.

Supplement: Supplementary file 1 [file DataSheet_1.pdf]
